# Modulatory and Toxicological Perspectives on the Effects of the Small Molecule Kinetin

**DOI:** 10.3390/molecules26030670

**Published:** 2021-01-28

**Authors:** Eman M. Othman, Moustafa Fathy, Amany Abdlrehim Bekhit, Abdel-Razik H. Abdel-Razik, Arshad Jamal, Yousef Nazzal, Shabana Shams, Thomas Dandekar, Muhammad Naseem

**Affiliations:** 1 Department of Biochemistry, Faculty of Pharmacy, University of Minia, Minia 61519, Egypt; moustafa_fathyy@yahoo.com (M.F.); amany_bkeet@mu.edu.eg (A.A.B.); 2 Department of Bioinformatics, Biocenter, University of Würzburg, Am Hubland, 97074 Würzburg, Germany; 3 Department of Regenerative Medicine, Graduate School of Medicine and Pharmaceutical Sciences, University of Toyama, Toyama 930-0856, Japan; 4 Department of Histology, Faculty of Veterinary Medicine, Beni-Suef University, Beni-Suef 62511, Egypt; abdelrazak.osman@vet.bsu.edu.eg; 5 Department of Biology, College of Science, University of Ha’il, Ha’il 2240, Saudi Arabia; imarshadjamal@gmail.com; 6 Department of Life and Environmental Sciences, College of Natural and Health Sciences, Zayed University, Abu Dhabi 144534, UAE; yousef.nazzal@zu.ac.ae; 7 Department of Animal Sciences, Faculty of Biological Sciences, Quaid-i-Azam University, Islamabad 45320, Pakistan; shubana.shams3@gmail.com

**Keywords:** cytokinin kinetin, modulatory effects, in vivo toxicity, A2a-R receptor

## Abstract

Plant hormones are small regulatory molecules that exert pharmacological actions in mammalian cells such as anti-oxidative and pro-metabolic effects. Kinetin belongs to the group of plant hormones cytokinin and has been associated with modulatory functions in mammalian cells. The mammalian adenosine receptor (A2a-R) is known to modulate multiple physiological responses in animal cells. Here, we describe that kinetin binds to the adenosine receptor (A2a-R) through the Asn253 residue in an adenosine dependent manner. To harness the beneficial effects of kinetin for future human use, we assess its acute toxicity by analyzing different biochemical and histological markers in rats. Kinetin at a dose below 1 mg/kg had no adverse effects on the serum level of glucose or on the activity of serum alanine transaminase (ALT) or aspartate aminotransferase (AST) enzymes in the kinetin treated rats. Whereas, creatinine levels increased after a kinetin treatment at a dose of 0.5 mg/kg. Furthermore, 5 mg/kg treated kinetin rats showed normal renal corpuscles, but a mild degeneration was observed in the renal glomeruli and renal tubules, as well as few degenerated hepatocytes were also observed in the liver. Kinetin doses below 5 mg/kg did not show any localized toxicity in the liver and kidney tissues. In addition to unraveling the binding interaction between kinetin and A2a-R, our findings suggest safe dose limits for the future use of kinetin as a therapeutic and modulatory agent against various pathophysiological conditions.

## 1. Introduction

Screening existing or recently discovered agents for alternative activities and a new therapeutic application has become an attractive approach [1,2,3,4,5,6]. Natural products-derived compounds exhibit various pharmacological effects and modulate, in vitro and in vivo, different signaling pathways involved in proliferation, migration, angiogenesis, apoptosis, oxidative stress, and inflammation-related disorders. Recently, many researchers began to investigate new biological activities for known or uncharacterized natural compounds. This is a new repurposing strategy to identify new therapeutic or prophylactic agents benefitting from the safety and apparent activities of these natural compounds [7,8,9,10,11,12]. One of the highlighted natural compounds which showed promising pharmacological activities in human cells are plant hormones such as cytokinins [13,14,15].

The small regulatory molecule N6-furfuryladenine (N6FFA) is commonly known as kinetin. It is a type of plant growth regulator, and belongs to the group of cytokinins (CKs) [16]. In plants, CK signaling operates through a two-components system (TCS), where the binding of CKs to the CHASE-domain of sensor histidine kinases (HKs) initiates a phosphorylation cascade involving histidine phosphotransfer proteins (HPTs) and B-type response regulators (B-ARRs) [17,18]. The B-type ARRs activate the expression of A-type response regulators (A-ARRS), which inhibit the signal of CKs in plants [17]. Similar to various other CK types, kinetin also binds to almost all known CKs receptors and causes analogous physiological responses in plants [17]. Understanding the signaling pathways of CKs in mammalian cells is still a *terra incognita*, as mammalian cells are devoid of the analogous TCS known from plants [19]. Despite this fundamental discrepancy, CKs get a lot of attention for their anti-ageing [20], neuro-protective [21,22], anti-cancerous [23], and anti-inflammatory [24] effects evident after tests in various types of mammalian cell lines.

In plants, kinetin binds to the CHASE-domain of Arabidopsis histidine kinase 4 (AHK4) and initiates a cascade of phosphorylation events to induce the expression of cytokinin response genes. In addition to many interesting functions that kinetin performs in mammalian cellular systems, the binding target sites of this important small molecule are not well elucidated until now. Only a few of the kinetin binding proteins (KPBs) have been studied so far. One of the putative KBPs is the adenosine receptor A2a-R, which is a drug target site for many cellular processes such as neuroprotection, cell proliferation, vascular regulation, as well as immunomodulatory functions [6,7,9]. Until now, only four types of adenosine receptors (A1R, A2aR, A2bR, and A3R) have been characterized. Among them, A1R and A3R belong to the group of G-coupled proteins that inhibit the adenylate cyclase-mediated production of the cAMP. In contrast, A2aR and A2bR belong to Go/Gs-coupled receptors that raise intracellular levels of cAMP and cause an anti-inflammatory response. Similar to adenosine and its analogs, which exert diverse anti-inflammatory effects such as the inhibition of TCR-mediated activation and cytokine production by T cells. One of the possible suggested mechanisms, which in this study was examined in silico and could explain the kinetin mediated effects in the mammalian cells is the binding of kinetin to A2a-R. The clear binding interactions observed *in silico*, together with the found pharmacological and protective effects suggest that the analogues modulatory functions in mammalian cellular systems are known from plants. One prominent previous study supporting our notion is the inhibition of the T lymphocyte activity via A2a-R activation by the plant hormone zeatin riboside [24]. Moreover, only the riboside form of kinetin has been shown to interact with A2a-R in invoking neuroprotection in a Huntington’s disease (HD) model [21]. However, a recent study demonstrated that kinetin in its native form can also protect HD-models [22]. Assessing the binding dynamics of kinetin for A2a-R with structural biology approaches helps better elucidate important residues for functional studies about kinetin as a potential modulator of diverse responses through the A2a-R receptor in mammalian cells.

As a first step before elucidating and applying the outcomes from the in silico modeling, it was important to examine the safety of using kinetin in an in vivo system and evaluate the safe, non-toxic doses which then can be scrutinized further for biological activity and therapeutic actions in future studies.

The in vitro effects of kinetin in terms of protection against oxidative stress have been extensively analyzed for the first time in a report by our group in various cellular systems with diverse potencies such as a human promyelocytic cell line (HL-60), a human keratinocyte cell line (HaCaT), an epithelial rat kidney cell line (NRK), and even in freshly isolated human lymphocytes [25,26]. It was demonstrated that kinetin concentrations below 100 nM can protect mammalian cells against genotoxicity induced cell death, as well as apoptosis [25]. In contrast, kinetin concentrations above 100 nM induce moderate genotoxicity, as well as cytotoxicity in the treated cells [10]. Likewise, the exogenous application of 1–10 µM concentrations of kinetin has shown no significant toxicity effects on the nerve cells [22]. More recently, it has been demonstrated that human promyelocytic HL-60 cells treated with less than 500 nM kinetin did not show a significant reduction in cell viability and genotoxicity [26]. However, higher kinetin concentrations beyond 500 nM induced both cytotoxicity and genotoxicity in the treated cells. Moreover, the H_2_O_2_ treated cells were protected from oxidative burst when simultaneously treated with a low concentration of kinetin (100 nM). However, higher kinetin concentrations (more than 500 nM) failed to rescue cells from the oxidative burst [25,26]. It is noteworthy to mention that all these previous studies pertinent to the rescuing effects of kinetin have been conducted on diverse cell lines. Despite these in vitro studies about the protective effects of kinetin against cellular toxicity, literature is scarce about the in vivo effects of kinetin. One prominent study in this context showed that there is no toxicity in mice administered kinetin intraperitonealy or orally at doses of 4.17 or 6.53 mg/kg [22]. Moreover, kinetin treatments furnished a visible improvement including curing the severe neurological conditions of Huntington’s disease [22].

To broaden the scope of kinetin as a future therapeutic agent against oxidative stress, as well as a modulatory agent, it is directly required to assess the in vivo toxicity of kinetin in an animal model. In the present study, we applied structural biology approaches to study the interaction between A2a-R and kinetin to find important binding residues for later functional studies and assays. We performed a metanalysis on the expression of the A2a-R gene across diverse anatomical parts including kidney and liver tissues. The present study investigated, for the first time the systemic effects of kinetin in adult male albino rats after the acute treatment with different doses of kinetin. Our study provides a baseline for the future use of kinetin as a potential therapeutic agent against cellular inflammatory responses and oxidative stress arising from various pathophysiological conditions.

## 2. Results and Discussion

### 2.1. The Interaction between Kinetin and A2a-R Receptor Provides Mechanistic Insights into the Kinetin Action

**Figure 1:**
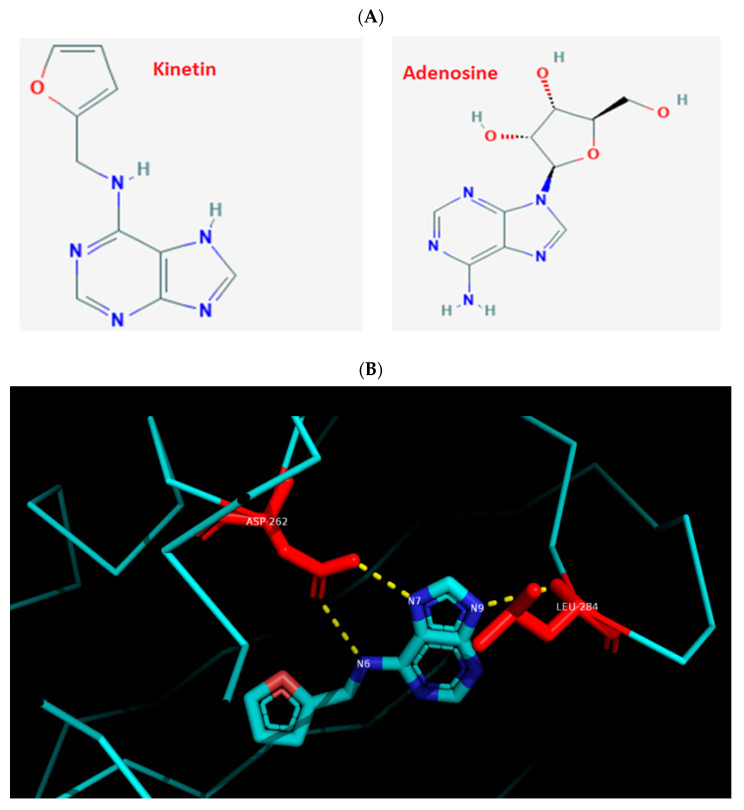

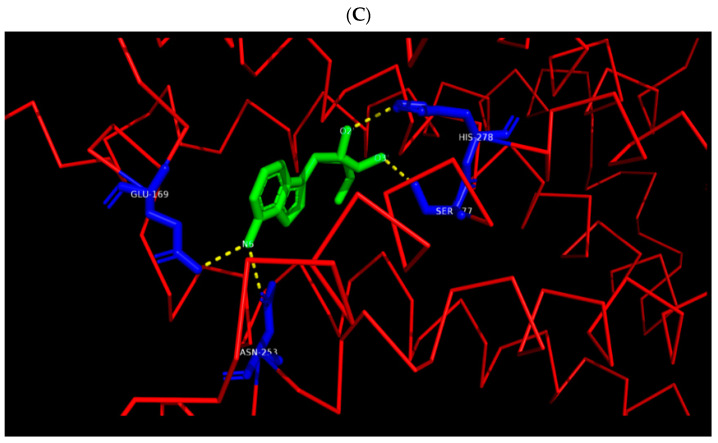
Visualization of the binding residues of AHK4 to kinetin and A2A-R to adenosine. (**A**) Chemical structure depiction for kinetin and adenosine derived from the PubChem database. (**B**) The 3t4s (AHK4) visualization in PyMol. AHK4 A chain residues Asp262 and Leu284 interact with kinetin. (**C**) The 2ydo (A2A-R) visualization in PyMol. The A2A-R residues Glu169, Asn253, Ser277, and His278 interact with adenosine.

**Figure 2:**
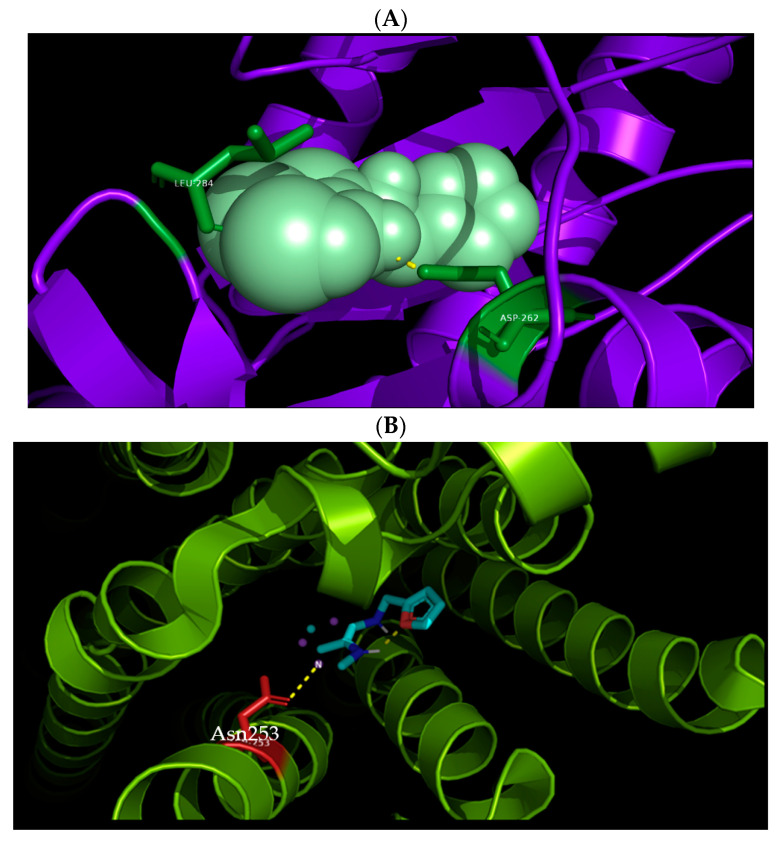
Docking of kinetin to *Arabidospsis* AHK4 and human A2A-R receptors. (**A**) Visualization of kinetin docked by our software to the AHK4 A chain in PyMol. Kinetin binds Leu284 and Asp262 residues of the AHK4 A chain as expected from the crystallography data. The calculated minimum binding energy state was −6.53 kcal/mol. (**B**) Visualization of kinetin docked to A2A-R in PyMol. Kinetin binds to A2A-R through the Asn253 residue. The calculated minimum binding energy state was at −4.29 kcal/mol.

Previous studies have demonstrated the anti-ageing, anti-inflammatory, immunomodulatory, and neuro-protective effects of kinetin in mammalian cells [20,21,22,23]. Despite the lack of understanding of its complete detailed biogenesis [22] or its targets in human cellular proteome and functional implications for mammalian cells [26], kinetin caught much attention recently for its potential therapeutic applications. One of the putative kinetin binding targets (KBP) is the adenosine receptor. Here, the binding of zeatin-riboside (cytokinin type, kinetin analogue) has been shown, for example, to inhibit the T lymphocyte activity via the adenosine A2a-R receptor activation [21]. Moreover, a riboside form of kinetin was shown to interact with A2a-R in mediating the neuroprotection in HD models [24]. Most of the CKs binding domains (CHASE-domain of histidine kinases) are studied in plants and microbial cells [18,19] and their binding sites in mammalian cells have not been systematically investigated. Similar to kinetin, adenosine is an adenine with added ribose sugar (Figure 1A) that binds to A2a-R, and so does the naturally occurring zeatin-riboside [21]. To get structural insights into the binding dynamics of kinetin to A2a-R, and as a first step to study the detailed action of kinetin at the molecular level before any experimental studies, we first compared and visualized the crystal structure of *Arabidospsis* AHK4 (plant cytokinin receptor) in the complex with kinetin along with the crystal structure of the human A2a-R complex with adenosine (Figure 1). We located the interacting residues of both these complexes: AHK4 A chain residues Asp262 and Leu 284 interact with kinetin (Figure 1B). Likewise, A2a-R residues Glu169, Asn253, Ser277, and His278 interact with adenosine (Figure 1C). Apparently, there are no common residues between *Arabidospsis* AHK4 and human A2a-R in their respective binding pockets for kinetin and adenosine. Then, we docked kinetin to the human A2a-R using the Autogrid and Autodock software. As a positive control, kinetin was also docked to the A chain of *Arabidospsis* AHK4. The visualization of kinetin docked to the AHK4 A chain shows that Leu284 and Asp262 are the residues through which kinetin binds to *Arabidospsis* AHK4 A with a minimum binding energy of −6.53 kcal/mol (Figure 2A). These are the same residues which are visualized in the crystal structure of AHK4 and kinetin complex (Figure 1B). These concordant results from docking, binding energy, and crystal structure validate the docking output and the notion that the adopted computational approach is working here correctly. In a previous study, we found multiple kinetin binding residues on the A2a-R receptor in a non-adenosine binding socket [26]. To get insight into the functional homology between kinetin and adenosine, we specifically focused on the adenosine binding sockets of the A2a-R receptor. Our docking analysis shows that kinetin binds to A2a-R with a minimum binding energy as −4.29 kcal/mol and the binding residue is Asn253 (Figure 2B). It is noteworthy to mention that the kinetin binding residue Asn 253 of A2a-R is one among the two residues through which adenosine binds to this well-known human receptor. From these analyses, we infer that there is a partial overlap between the binding dynamics of kinetin and adenosine for the A2a-R receptor.

Future studies focusing on A2a-R over-expressing cells or using an A2a-R knockout mouse, are needed and planned to prove that kinetin interacts with A2a-R and to confirm that similar to adenosine, it in fact, serves as an agonist in anti-inflammatory responses in mammlian cells. If this is true, the Asn 253 residue of A2a-R will further determine the molecular basis of kinetin actions in mammalian cells. Our structural biology analysis provides a lead that kinetin mediated anti-inflammatory responses in cells should involve the A2a-R receptor. In addition to maybe direct immunomodulatory effects, we further speculate that kinetin may have profound effects on the maturation of primary immune cells into the B cell, T cell, and even macrophages. To support this notion, it is known that the treatment of kinetin delays senescence by increasing cell survival under normal conditions and even during the condition of oxidative burst [25]. Moreover, the anti-senescent properties of kinetin and its action in delaying cell death should favor the spread of the pathogens in the infected tissue as well as, increase the maturation time of immune cells such as macrophages required for clearing the attacking pathogen or the cancerous cells. In support of this notion, delaying the immune cell maturation can also be hijacked by cytokinin secreting human pathogens such as *Mycobacterium tuberculosis* [27] and evade active immune defenses [19]. These and the similar hypothesis pertaining to the immunomodulatory roles and other pharmacological actions of kinetin merit a detailed further investigation.

**Figure A1:**
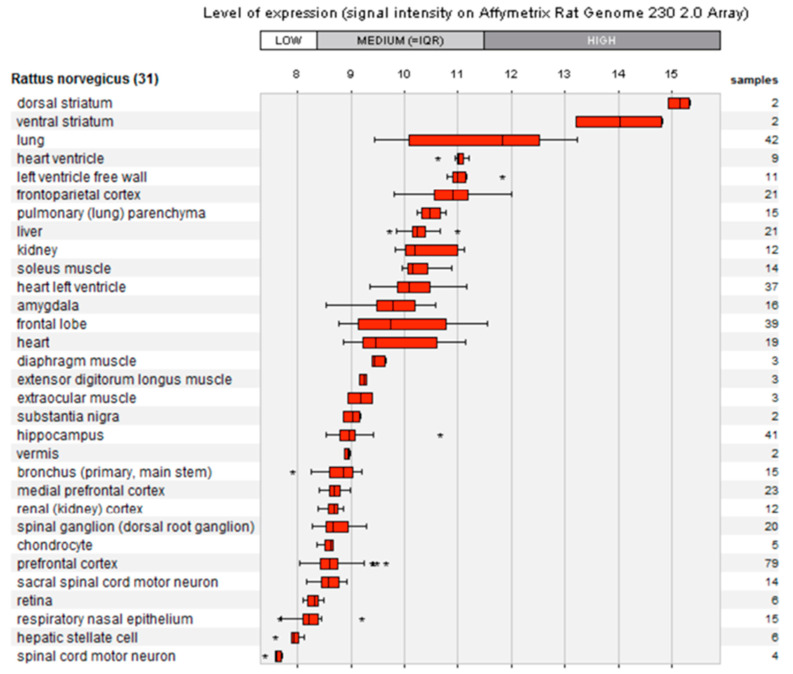
Metanalysis of the A2a-R gene expression in various tissues of *Rattus Norvegicus*. GENEVESTIGATOR (https://genevestigator.com) mined gene expression datasets for 31 anatomical parts showing the Affymetrix based intensity for one gene (A2aR). The expression is shown as an average of the signal intensity in samples (shown on the right side of the graph) upon diverse treatments. The liver and kidney samples show a moderate level of upregulation in all the tested samples. The level of expression is shown in a log-scale. All data shown are available at the database. * *P* < 0.05, *** *P* < 0.001.

Next, we performed a metanalysis on the regulation of the mammalian A2a-R gene across anatomical parts of the rat by mining the GENEVESTIGATOR database (Figure A1). We found that 31 different tissues of the rat overexpress the A2a-R gene over diverse genetic, pharmacological, and environmental perturbations with low, middle, and high expression thresholds. In order to collect a systemic anti-inflammatory response of kinetin via the ubiquitous regulation of A2a-R across multiple tissues types (Figure A1), a systemic study of the effect of kinetin will be highly warranted. Renal and hepatic tissues being at the forefront of detoxification processes are always prioritized for the toxicological analysis. As shown in our metanalysis, A2a-R is highly regulated in liver and kidney organs under 21, as well as 12 different treatments, respectively (Figure A1). Therefore, a systemic toxicological analysis of the general body fluids such as blood plasma, and renal and hepatic tissues under various doses of kinetin for pharmacological (anti-inflammatory) effects through the A2a-R receptor or similar targets is highly desirable. Therefore, we performed a detailed toxicological analysis to provide a baseline for the future pharmacological applications of kinetin to act as protective under acute harmful external conditions.

### 2.2. Systemic Effects of Kinetin in Albino Male Rats in Terms of Morbidity and Mortality

We previously studied the protective effects of kinetin against oxidative stress under genotoxic, as well as cytotoxic conditions in a variety of cell lines [25,26]. Almost all these studies have been conducted on cell lines through in vitro assays. Despite the experimental convenience that in vitro studies offer, these are unable to provide systemic insights into the toxicity, vitality, and efficacy of the administered bioactive compound to the cell culture. To harness the therapeutic potential of kinetin, we conducted an in vivo study after injecting various doses into adult male albino rats. Owing to their similarity with the human digestive system and having a food composition similar to that typically eaten by humans, male albino rats are very suitable for experimental replicates and data reproducibility due to their small size and ease in handling in toxicological examinations.

In this study, we assessed the acute toxicity (14 days) of low and high concentrations of kinetin in adult male albino rats. We used a range of kinetin doses such as 0, 0.25, 0.5, 1, and 5 mg/kg. In our previous study [25,26], we observed a biphasic response of protection and vulnerability in cell lines after the kinetin treatment with low (below 500 nM) and high (above 500 nM) concentrations. Taking that into account, we assessed the mortality rate of the treated animals. We did not observe any mortality (0%) or morbidity (0%) in the treated animals (all together 30 animals). Furthermore, we observed the treated animals for physical changes and daily activities during the 14 days treatment period with both high and low kinetin doses. We did not observe any visible changes in the physical activities of the treated rats. There are no previous reports on the correlation between the toxicological assessment of increased cytokinin concentrations in mammals. However, higher cytokinin concentrations led to the accumulation of antimicrobial compounds in plants that can kill plant pathogens [28]. Similarly, the increased production of cytokinin in *Mycobacterium tuberculosis* increased bacterial hypersensitivity [27]. Likewise, a higher cytokinin containing plant extracts when fed to insect larvae, caused twitching and thrashing activities in comparison to the insects fed with non-transgenic control leaves [29]. The lack of adverse effects of kinetin in the treated rats particularly at higher doses can be attributed to the process of detoxification that may occur at the organismal level to neutralize the toxicity of kinetin.

### 2.3. Kinetin Mediated Toxicity and the Serum Level of Metabolites and Enzymes Activity

**Figure 3:**
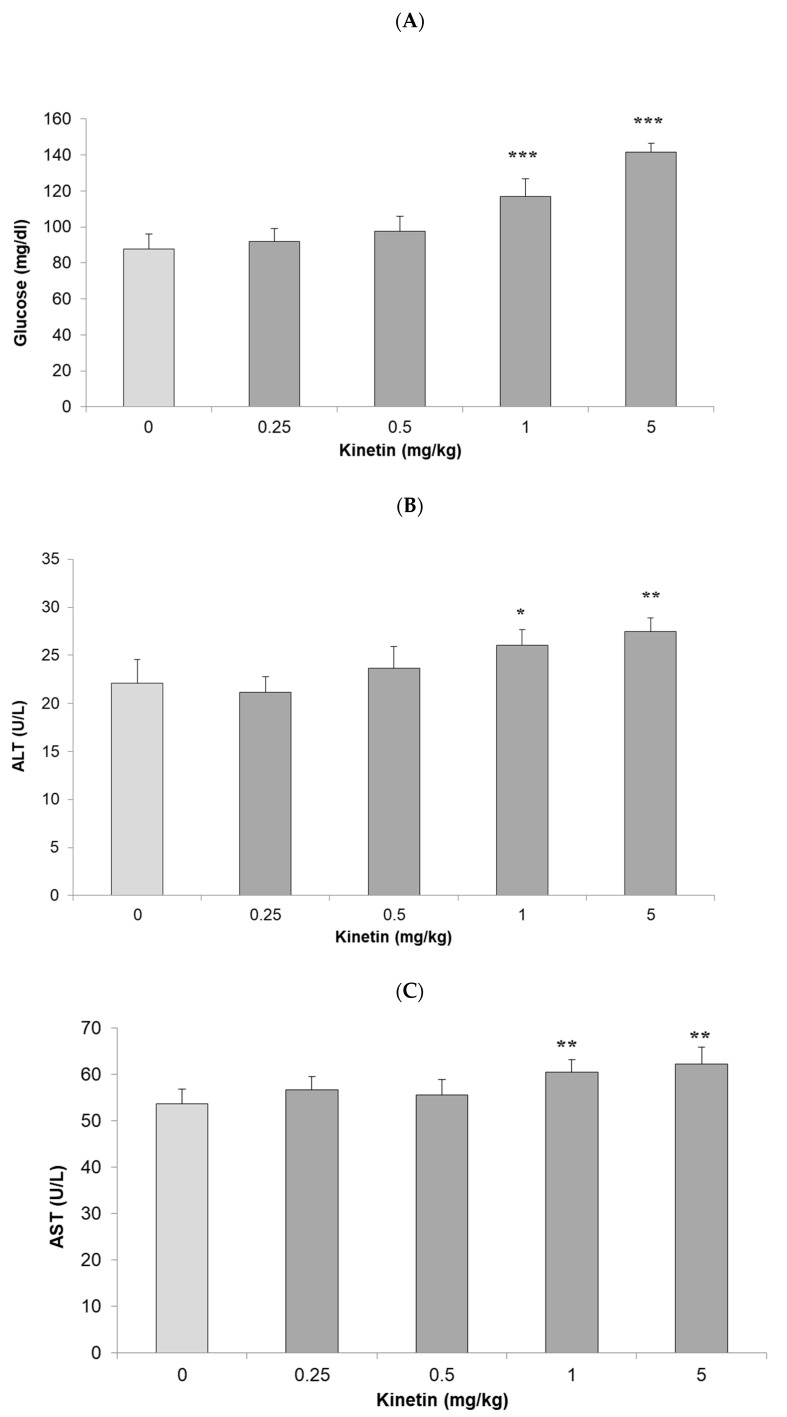

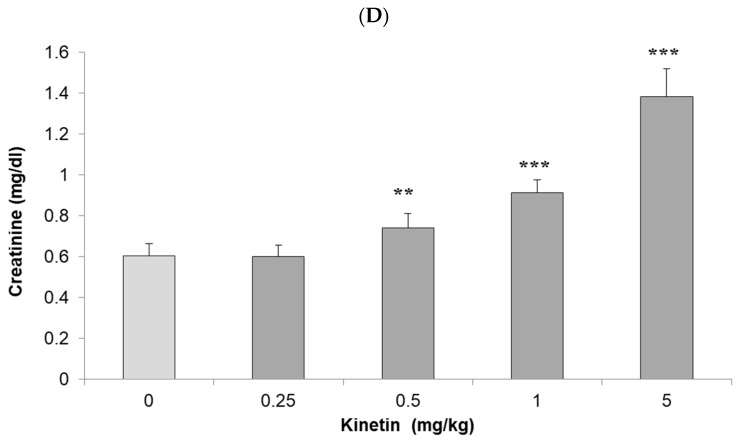
Biochemical investigation of kinetin effects in rats. Effect of kinetin on serum levels of (**A**) glucose, (**B**) alanine transaminase (ALT), (**C**) aspartate aminotransferase (AST) and (**D**) creatinine when rats were treated with different kinetin doses (0, 0.25, 0.5, 1 and 5 mg/kg). Data represented as mean ± SD (n = 6 rats). Significant differences were analyzed by Student’s *t*-test, whereby * *P* < 0.05, ** *P* < 0.01, *** *P* < 0.001 is denoted compared to the control group.

The liver and kidney are among the most vital and central detoxification organs that are affected by the toxicity of compounds. Their damage can lead to severe complications such as renal failure, hepatic cirrhosis, or even death. To assess the systemic effects of kinetin, we collected serum, liver, and kidney samples of the treated rats and control groups. Then, we assessed the level of different markers such as glucose level, alanine transaminase (ALT), aspartate aminotransferase (AST), and creatinine in the serum of the treated rats to determine the systemic (toxic) effects of various doses of kinetin. The lower doses of kinetin such as 0.25 and 0.5 mg/kg body weight did not increase the level of glucose as compared to the control groups (Figure 3A). However, higher doses such as 1 and 5 mg/kg caused a significant increase in the serum glucose level. Likewise, we did not observe any significant difference in the serum ALT and AST enzymes for treated rats in comparison to the control groups (Figure 3B,C). However, similar to the serum glucose level, we also noticed a significant increase in the activity of both these liver enzymes in the serum of the 1 and 5 mg/kg kinetin treated rats. To get insight into the impact of the kinetin treatment on kidney functions, we determined the levels of creatinine in the serum of treated and control groups. Unlike serum glucose and liver functional enzyme markers, we found a significant increase in serum creatinine levels at 0.25 mg/kg kinetin dose and the difference was even more pronounced at higher doses of the kinetin treatment (Figure 3D). Taken together, these results signify a dose dependent response of kinetin such that lower doses have no toxic effects on renal and hepatic functions. However, higher kinetin doses lead to potential malfunctioning in the activities of both these vital organs. In comparison to the liver, the kidney samples showed a sign of toxicity even at 0.25 mg/kg body weight of the kinetin treatment. The early appearance of toxicity in the kidney tissue shows its differential response to toxicity in comparison to the liver tissue, which better withstands toxic effects owing to the detoxification mechanisms it applies in the routine. These results further substantiate the validity of our previous findings [25,26] of the in vitro effects of kinetin on treated cell lines, where higher kinetin doses showed genotoxic and cytotoxic effects on the cells and lower doses further protected cells against the effects of genotoxic as well as cytotoxic agents.

### 2.4. Hepatic and Renal-Histopathological Examination of the Kinetin Treated Rats

**Figure 4:**
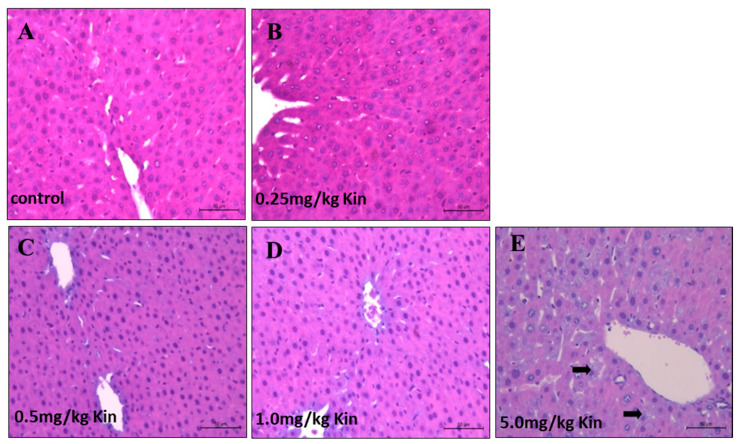
Histopathological examination of kinetin effects on rat livers. Shown are photomicrographs with an H&E stain (200× magnification) of livers from adult male albino rats. (**A**) The control group showed a normal liver with a central vein and normal hepatocytes. (**B**) The 0.25 mg/kg kinetin treated group showed a normal liver tissue. (**C**) The 0.5 mg/kg kinetin treated group showed a normal liver tissue. (**D**) The 1 mg/kg kinetin treated group showed a normal liver tissue. (**E**) The 5 mg/kg kinetin treated group with a degree of degeneration in a few hepatocytes (arrow).

**Figure 5:**
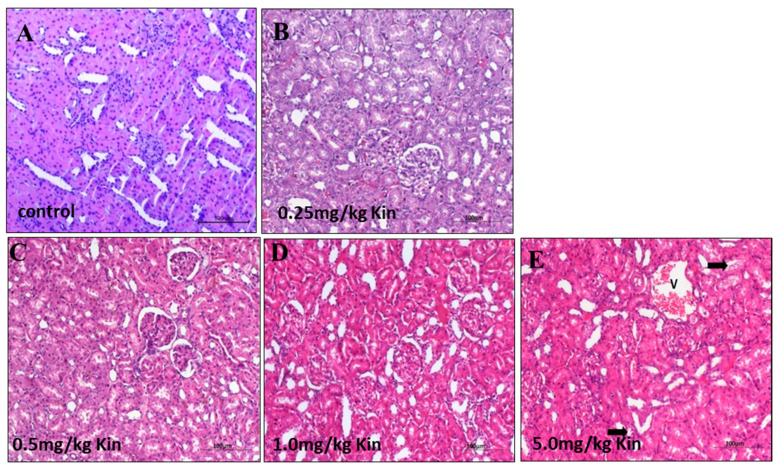
Histopathological examination of kinetin effects on rat kidneys. Shown are photomicrographs with an H&E stain (200× magnification) of kidneys from adult male albino rats. (**A**) The control group showed a normal renal tissue with normal renal corpuscle and tubules. (**B**) The 0.25 mg/kg kinetin treated group showed a normal renal tissue. (**C**) The 0.5 mg/kg kinetin treated group showed a normal renal tissue. (**D**) The 1 mg/kg kinetin treated group showed a normal renal tissue. (**E**) The 5 mg/kg treated kinetin group showed normal renal corpuscles, the renal glomeruli and tubules showed a mild degree of degeneration (arrow), as well as congestion of the renal blood vessels (V).

To get further insights into the kinetin mediated dose-dependent toxicity response, we performed an histopathological examination for liver and kidney tissues of the kinetin treated rats. According to our analysis, the photomicrograph of liver in rats showed a normal liver with central vein and normal hepatocytes similar to the control groups for 0.25, 0.5, and 1 mg/kg kinetin treatments (Figure 4A–D). However, 5 mg/kg of the kinetin treatment caused a degree of degeneration in a few of the observed hepatocytes (Figure 4E). Likewise, we also examined kidney histopathological sections and found that lower kinetin doses such as 0.25, 0.5, and 1 mg/kg showed a normal renal tissue with normal renal corpuscle and tubules such as control rats (Figure 5A–D). However, 5 mg/kg of the treated kinetin group showed normal renal corpuscles, but a mild degree of degeneration was observed for the renal glomeruli and tubules as compared to the control group (Figure 5E). Together, our histopathological findings indicate that the kinetin treatment caused mild toxicity and structural degradation in both hepatic and renal tissues at higher kinetin concentrations, whereas lower doses have no malfunctioning impact on the integrity of the challenged rats. Thus, our data from the biochemical analysis (Figure 3) are clearly corroborated by our histopathological analyses (Figure 4 and Figure 5) about the differential response of rats to low and higher kinetin doses.

## 3. Materials and Methods

### 3.1. Docking of Kinetin to the A2a-R Receptor

A local pairwise sequence alignment of AHK4 and A2A-R sequences was performed using the EMBOSS Water online tool. From the protein data bank (PDB), crystal structures of *Arabidopsis* AHK4 in complex with kinetin and in the human A2a-R receptor with adenosine bound were obtained with accession codes 3T4S and 2YDO, respectively. For a structural alignment of the AHK4 A chain and A2a-R we used the PyMol software. The crystal structure of AHK4 in complex with kinetin was retrieved from the PDB database. We used PyMol to visualize AHK4 residues interacting with kinetin. Likewise, the crystal structure of A2a-R was obtained in complex with adenosine and visualized by using PyMol. Eventually, kinetin was docked first to AHK4 and then to A2a-R using AutoGrid and AutoDock softwares. The graphical user interface Autodock tools (ADT) were also utilized. The conversions of the files were performed using the Open Babel software.

### 3.2. Experimental Animals

Kinetin was obtained from Sigma-Aldrich (Dorset, Germany), if not mentioned, all other chemicals or solvents were purchased from Santa Cruz Biotechnology (Heidelberg, Germany). This study was conducted on adult male albino rats (weight 150–180 g) and approved by the Research Ethics Committee for Animal Experimentation, Department of Pharmacology and Toxicology, Faculty of Pharmacy, Minia University, Egypt (project code no. 54/2019). Rats were housed and bred under standardized conditions in the pre-clinical animal house. They were kept in mesh-bottomed stainless-steel cages (six per cage), fed a standard diet, and allowed free access to drinking water. The animals were acclimatized to the environment for 1 week before commencement of the experiments. All the conditions were carefully maintained to minimize animal suffering.

### 3.3. Experimental Design

After an adaptation period of 7 days, rats were randomly divided into five groups of six animals each. Treatments were then carried out according to the following design Group 1: Served as the normal control group, which received the saline only for 14 days. Group 2, 3, 4, and 5: Received different doses of kinetin (0.25, 0.5, 1.0, and 5.0 mg/kg body weight, respectively) for 14 days through an intraperitoneal injection.

### 3.4. Biochemical Assays

Freshly collected blood samples after scarification were centrifuged at 3000 rpm for 10 min. The obtained clear serum was used for measuring the levels of glucose, ALT, AST, and creatinine using commercially available colorimetric assay kits (Sigma-Aldrich), according to the standard procedures [30].

### 3.5. Histopathological Examination

All the animals were sacrificed, by the end of the experiment the liver and kidney were collected and fixed in 10% of neutral buffered formalin. The excised organs were routinely processed dehydrated in ascending grades of ethanol and embedded in Paraffin wax. Then, the obtained blocks were sectioned at 4 to 5 mm thickness. The obtained tissue sections were collected on clean and dry glass slides, deparaffinized, and stained with the Hematoxylin and Eosin stain [31]. Then, the sections were examined and observed under a light microscope at 100, 200, and 400× magnification (Leika DMRBE, Germany) for the determination of pathological alterations.

## 4. Conclusions and Outlook

In conclusion, our results from the structural biology analysis identified similar binding residues and binding modes between kinetin and A2a-R, as a first intriguing receptor target suggested by the in silico analysis. Such interactions can mediate the observed regulatory actions of kinetin into mammalian cells.

Our immunohistochemistry analysis hinted on the acute toxicity of rats only for doses of 5 mg/kg kinetin or higher, which is supported by a previous study (from a neurological point of view) where the intraperitoneal administration of 4.17 mg/kg of kinetin better protected the HD mouse model from severe neurological conditions without inducing toxicity in the treated mice [22]. Likewise, when administered at 5 mg/kg, kinetin significantly inhibited the apoptosis and promoted the proliferation of splenic lymphocytes. Moreover, kinetin did effectively enhance the immune power of aging rats and slow down the aging process [32]. Furthermore, it was observed that the treatment with kinetin, at doses of 4 and 6 mg/kg body weight, may lower the risk of thromboembolic-related disorders and was effective in reducing the mortality of acute pulmonary thromboembolism in mice [33]. It is challenging to compare the body weight in vivo kinetin administration doses to the in vitro kinetin concentrations. The latter are in nano-molar or micro-molar concentrations, as we previously demonstrated in in vitro studies using cell lines [25,26]. However, previous in vivo studies (although somewhat fragmentary) [32] demonstrated that under various conditions, the 5 mg/kg body weight kinetin dose retains pharmacological effects. Thus, our study provides a first baseline for future studies harnessing the effects of kinetin as a potential therapeutic agent [34] in mitigating oxidative stress and potentially promoting beneficial modulatory responses of kinetins. However, there are many open questions regarding the fundamental molecular understanding of kinetins. For instance, how kinetin is internalized and metabolized by mammalian cells [35], and what are the other drug target sites in mammalian cells where kinetin binds and invokes both anti-inflammatory and cytotoxic effects in various concentrations [26]. Therefore, a holistic approach of assessing time-resolved transcriptomes and metabolomes (including further lipids such as cholesterol and triglycerides) will be required to dissect the molecular mechanisms of kinetin biology in mammalian cells. Moreover, large scale, small, molecule-protein interaction studies [36] will also help shed light on the kinetin regulated pathways as drug target sites in mammalian cells. The outcome of these analyses will further unleash the potential of kinetin as a next generation of pharmaceutical agents [37,38] with modulatory effects against pathophysiological conditions arising from pathogen infection, diabetes, or cardiovascular complications.

## Data Availability

All data are fully available and included in the manuscript and Appendix A.

## Sample Availability

Samples of the compounds are not available from the authors.
